# Lipomatous Hypertrophy of the Interatrial Septum (LHIS) a Biomarker for Cardiovascular Protection? A Hypothesis Generating Case–Control Study

**DOI:** 10.3390/jcdd12080301

**Published:** 2025-08-04

**Authors:** Pietro G. Lacaita, Valentin Bilgeri, Fabian Barbieri, Yannick Scharll, Wolfgang Dichtl, Gerlig Widmann, Gudrun M. Feuchtner

**Affiliations:** 1 Department Radiology, Innsbruck Medical University, 6020 Innsbruck, Austria; pietro.lacaita@i-med.ac.at (P.G.L.); yannick.scharll@i-med.ac.at (Y.S.); gerlig.widmann@tirol-kliniken.at (G.W.); 2 Department Cardiology, Innsbruck Medical University, 6020 Innsbruck, Austria; valentin.bilgeri@tirol-kliniken.at (V.B.); wolfgang.dichtl@tirol-kliniken.at (W.D.); 3 Department of Cardiology, Angiology and Intensive Care Medicine, Deutsches Herzzentrum Charite, 10117 Berlin, Germany; fabian.barbieri@dhzc-charite.de

**Keywords:** imaging, computed tomography, cardiovascular risk, epicardial adipose tissue, interatrial septum, coronary artery disease, atherosclerosis

## Abstract

**Background:** While epicardial adipose tissue (EAT) is a known predictor of adverse cardiovascular outcomes, lipomatous hypertrophy of the interatrial septum (LHIS) is composed of metabolically active fat such as brown adipose tissue, which may exert a different effect. This study investigates the coronary atherosclerosis profile in patients with LHIS using CTA, compared with a propensity score-matched control group. **Methods:** A total of 142 patients were included (*n* = 71 with LHIS and *n* = 71 controls) and propensity score-matched for age, gender, BMI, and the major CV risk factors (matching level, <0.05). CTA imaging parameters included HRP, coronary stenosis severity (CADRADS), and CAC score. **Results**: The mean age was 60.9 years +/− 10.6, there were nine (6.3%) women, and the mean BMI is 28.04 kg/m^2^ +/− 4.99. HRP prevalence was significantly lower in LHIS patients vs. controls (21.1% vs. 40.8%; *p* < 0.011), while CAC (*p* = 0.827) and CADRADS (*p* = 0.329) were not different, and there was no difference in the obstructive disease rate. There was no difference in lipid panels (cholesterol, LDL, HDL, TG) and statin intake rate. **Conclusions:** HRP prevalence is lower in patients with LHIS than controls, while coronary stenosis severity and CAC score are not different. **Clinical relevance:** LHIS may serve as imaging biomarker for reversed CV risk.

## 1. Introduction

Coronary heart disease remains the primary cause of mortality in both Europe and the United States, underscoring the critical importance of precise risk stratification aimed at mitigating adverse outcomes [1].

Conventional cardiovascular (CV) risk scores, such as the ASCVD-Pooled Cohort Equations [2] or the European SCORE model [3], have shown limitations in calibration and discrimination, especially in specific subgroups. The updated systematic coronary risk estimation 2 (SCORE2) algorithm, endorsed by the European Society of Cardiology, improves upon previous models by incorporating competing risks and recalibration. Nonetheless, even SCORE2 shows only moderate discriminatory performance (with the C-index ranging from 0.67 to 0.81 depending on age group and cohort), which underscores the need for more precise imaging biomarkers to refine individual risk stratification.

Cardiac computed tomography angiography (CTA) imaging biomarkers improve CV risk stratification: The coronary artery calcium score (CAC), a quantitative measure of the total calcifying plaque burden [4], the coronary stenosis severity [5,6] from coronary computed tomography (CTA), and the coronary plaque burden, such as total plaque volume (TPV) [7] and plaque phenotypes, add incremental prognostic values to conventional risk scores. High-risk plaque (HRP) features, for example, can identify patients at the highest risk, with up to a four-fold increase in MACE events [8]. Emerging imaging parameters, such as epicardial adipose tissue (EAT) and pericoronary adipose tissue (PCAT), further enhance CV risk stratification [9,10].

Lipomatous hypertrophy of the interatrial septum (LHIS) is a special compartment of the EAT, characterized by distinct CT density values suggestive of a more benign fat phenotype such as brown fat or paracardial fat [11,12]. Unlike white adipose tissue, brown adipose tissue (BAT) is metabolically active and has been associated with improved lipid metabolism and reduced cardiometabolic risk [13,14]. In previous studies, LHIS accounted for up to 4.3% of the EAT [11,12]. Therefore, LHIS may act as “positive” CV risk biomarker for reversed cardiovascular risk; however, its relationship with CAD, as assessed by CTA, has not yet been investigated.

Therefore, the objective of our study was to compare the CAD profile, assessed by coronary computed tomography angiography (CTA), including coronary stenosis severity (CADRADS) and high-risk plaque (HRP) phenotypes, total plaque burden, and the coronary artery calcium score (CAC), in patients with LHIS compared to a propensity score-matched control group without LHIS.

## 2. Materials and Methods

### 2.1. Study Cohort

Patients who underwent coronary CTA between 01/2010 and 10/2021 for clinical indications according to ESC guidelines [15] were included in our retrospective study registry. Patients with suspected coronary artery disease based on their clinical presentation (e.g., atypical or typical chest pain, low-to-intermediate pre-test probability), risk profile, and other test findings were included. Institutional review board (IRB) approval for the database was obtained.

### 2.2. Inclusion Criteria

(1) Lipomatous hypertrophy of the interatrial septum (LHIS): This is defined as a mass-like lipomatous tissue infiltration of the entire interatrial septum from cranial to caudal, sparring the fossa ovalis, with a “dumb-bell” configuration with >than 5 mm width.The maximal width was identified on axial images and measured with a digital caliper. The maximal length was defined as the distance from the anterior to the posterior borders.
(2) Control group (CR): Patients were propensity score-matched for age, body mass index (BMI), and the major cardiovascular risk factors (smoking, arterial hypertension, positive family history, dyslipidemia, and diabetes), with a matching level of 0.05. Further, the groups were balanced for the co-morbidity COPD. All CT scans were reviewed for the absence of LHIS by an experienced observer (a board-certified radiologist with >10 years of experience in cardiac CT).

Exclusion criteria: Prior coronary artery bypass grafting (CABG), or referrals for other clinical indications such as structural heart disease intervention planning (TAVI planning).

Computed tomography (CT) and coronary artery calcium (CAC) score: A non-contrast ECG-gated CT scan (detector collimation 2 × 64 × 0.6 mm; 120 kV; image reconstruction 3 mm slice width, increment 1.5) and prospective ECG-gating was performed. The Agatston Score (AU) [16] was calculated with automated software (Cardiac CT, SyngoVIA, Siemens Healthineers, Erlangen, Germany) as a quantitative measure for calcified plaque burden.

Coronary CTA was performed with a 128-slice dual-source CT (Somatom Definition FLASH or DRIVE, Siemens: detector collimation 2 × 64 × 0.6 mm, z-flying spot, and rotation time 0.28 s). Prospective ECG-triggering in the diastolic phase was applied to patients with regular heart rates < 65 bpm, and retrospective ECG-gating was applied to those with >65 bpm and irregular rates. An iodine contrast agent (Iopromide, Ultravist 370™) was injected intravenously (flow rate: 4–6 mL/s + 40 cc saline; contrast volume: 65–120 mL). Axial images were reconstructed with a 0.75 mm slice width (increment 0.4/medium-smooth kernel B26f) during the best diastolic and systolic phases. Curved multiplanar reformations (cMPR) and oblique interactive MPR using client server-based 3D post-processing software (CardiacCT, SyngoVia^TM^, Siemens Healthineers) were generated, and the following CTA parameters measured:

(1) Coronary stenosis severity was scored visually according to CAD-RADS^TM^ [15] classification as minimal (CADRADS-1) < 25%, mild (CADRADS-2) 25–49.9%, moderate (CADRADS-3) 50–69.9%, severe (CADRADS-4) ≥ 70–99%, and occluded 100% (CADRADS-5) on a per-coronary segment-base (AHA-modified-17-segment classification), assisted by quantitative stenosis measurement using curved multiplanar reformations (MPR).
(2) For coronary plaque phenotypes, high-risk-plaque (HRP) analysis was carried out according to the CADRADS [16]. Low attenuation plaque (LAP) was defined as a hypoattenuating lesion with <150 HU. CT-density was screened with the “pixel lens,” and the lowest HU were recorded. LAP < 30 HU was defined as lipid-rich necrotic core [8] and LAP < 60 HU as fibrofatty plaque. A napkin-ring sign was defined as a lesion with an outer high-density rim with an inner hypodense area [16]. Spotty calcification was defined as a calcification of less than 3 mm size. Positive remodeling was defined as a remodeling index (RI) of >1.1. A patient was labeled as “HRP” if a minimum of two criteria were present, and if at least one LAP < 30 HU or LAP < 60 HU was present per patient. Coronary CTA analysis was performed by one highly experienced reader (>5–10 years cardiac CT). The number of HRP criteria per patient was recorded (n = 2, n = 3, or n = 4), and the sum of HRP was calculated for each group. HRP analysis was repeated by a second observer with 3 years cardiac CT experience after 1 year, and by the same observer (with 10 years of cardiac CT experience) another 1 year.
(3) Coronary plaque burden was classified according to the CADRADS 2.0 [16]. P-classification was as follows: P0 (no plaque); P1 (mild plaque burden, segment involvement score (SIS) < 2 = 1–2 vessels with mild amount of plaque); P2 (moderate, SIS 3–4); P3 (severe, SIS 5–7); and P4 (extensive plaque CAC > 1000 AU, SIS > 8).
(4) Non-coronary CTA parameters: CT density of LHIS was measured. A circular region-of-interest (ROI) was placed in the area of maximal LHIS width, and CT density (HU) was measured. LHIS width and length were also measured.

Clinical data collection: Conventional cardiovascular risk factors (CVRF) were collected and defined according to the standardized European Society of Cardiology (ESC) criteria [3,17,18,19] arterial hypertension (systolic blood pressure (BP) > 140 mmHg or diastolic BP > 90 mmHg); dyslipidemia; positive family history (myocardial infarction (MI)); sudden cardiac death in an immediate male relative < 55 years or female < 65 years; smoking status (active: current or quit less than 6 months before coronary CTA examination and former); and diabetes. Medication potential affecting coronary artery disease was collected (statins). The SCORE2, according to ESC [3], was calculated for both the LHIS group and controls.

Cardiovascular outcome data collection: Cardiovascular outcomes were collected from electronic hospital charts. Invasive coronary angiography (ICA) results were recorded, and outcomes were defined as less or more than 50% stenosis. Coronary revascularization procedural data (defined as percutaneous coronary intervention (PCI) or coronary artery bypass grafting) were retrieved. Major cardiovascular events (MACE) were defined as death, myocardial infarct, and acute coronary syndromes (ACS), with both NSTEMI and STEMI ACS recorded.

Statistical methods: Statistical analysis was performed using SPSS™ software (IBM, V25.0, SPSS Inc., Chicago, IL, USA). A propensity score matchmaking model was applied to reduce the possibility of selection bias and confounding. Accordingly, a binary regression was calculated, including age, body mass index, and the major cardiovascular risk factors (smoking status, arterial hypertension, positive family history, dyslipidemia and diabetes). The derived propensity score was then utilized for matchmaking using a 1:1 nearest neighbor matchmaking process without replacement, with a matching tolerance of 0.05. Quantitative variables are expressed as means ± standard deviation (SD) or as median (interquartile range, IQR), and categorical variables as absolute values and percentages. The chi-square test was applied to test for differences in categorical binary data (e.g., CVRF, HRP prevalence), and the Fisher’s exact test was applied in cases of less than 5 counts between the 2 groups (LHIS and controls), and the independent t-test for normally distributed data (age, BMI) and the Mann–Whitney U for non-normally distributed data (CAC, CADRADS stenosis severity and total plaque burden (P0-4) visual classification) were also applied. Inter- and intraobserver variability for HRP detection was calculated with weighted Cohen’s kappa. 

## 3. Results

**Figure 1:**
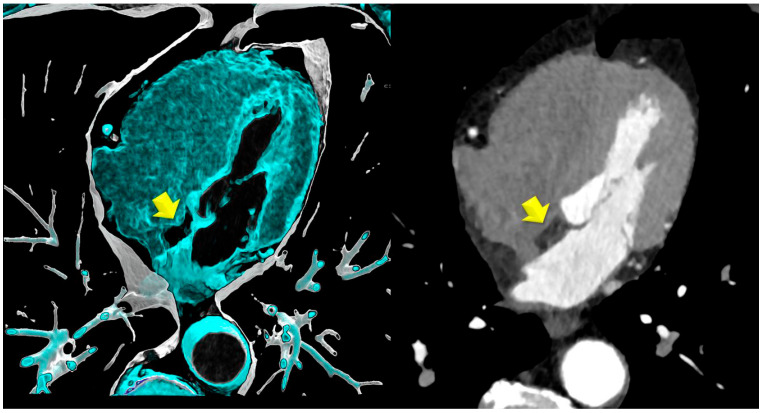
Lipomatous hypertrophy of the interatrial septum (LHIS) by CTA (yellow arrow). Left: volume rendering technique (VRT), thin slice reconstructions; right: multiplanar reformation (MPR), axial images.

The control group was selected from our retrospective study cohort, consisting of 603 patients, and the LHIS cohort was selected from our LHIS database, consisting of 195 patients (Figure 1).

Finally, 142 patients were propensity score-matched (*n* = 71 controls and *n* = 71 patients with LHIS). The mean age was 60.9 years ± 10.6, and nine (6.3%) were women. The mean body mass index (BMI) was 28.04 kg/m^2^ ± 4.99.

The patient profile and CTA results for the matched cohorts are shown in Table 1. The proportion of women was low, amounting to five (7%) of the patients with LHIS and four (5.6%) of controls (*p* = 0.999). BMI did not differ between the patients with LHIS and the controls (*p* = 0.096). The rates of AFIB (9.8% vs. 16.9%, *p* = 0.324) and COPD were low (7% vs. 5.6%, *p* = 0.999), and there were no difference between LHIS and controls. Three patients in the LHIS group had prior PCI /Stents; none (0%) of the controls did. There were no statistically significant differences in major cardiovascular risk factors (CVRFs), statin use, or lipid profiles.

**Figure 2:**
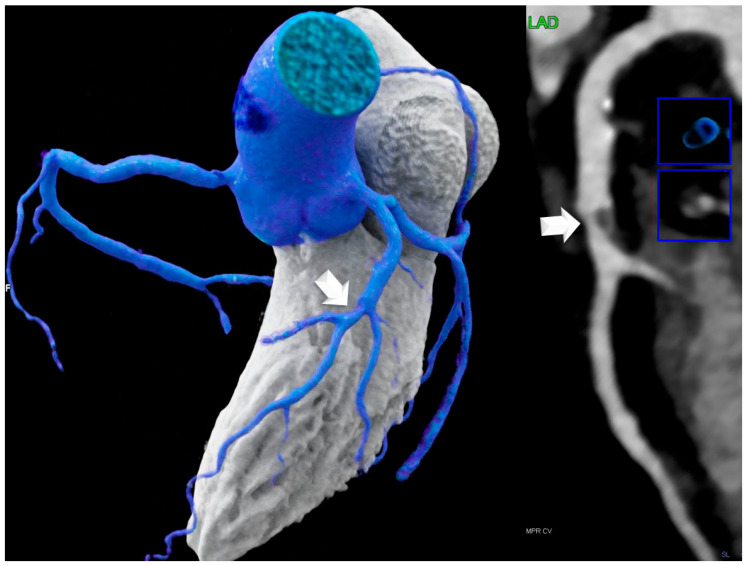
A 57-year-old male with a non-calcified lesion in the mid LAD with high-risk plaque (HRP) (white arrows and blue boxes) features (LAP 16- 31 HU, positive remodeling) from the control group without LHIS. He had two CVRF and >50% stenosis (CAD-RADS 3).

**Figure 3:**
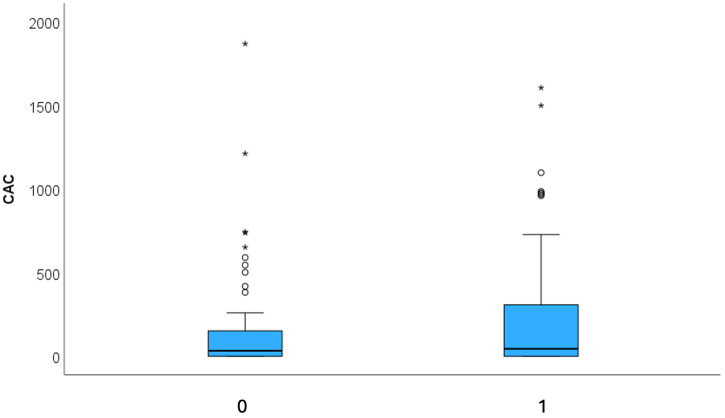
Comparison of atherosclerosis features by CTA between LHIS and controls: There was no difference in the coronary artery calcium score (CAC) in group 1 = LHIS vs. 0 = controls.

**Figure 4:**
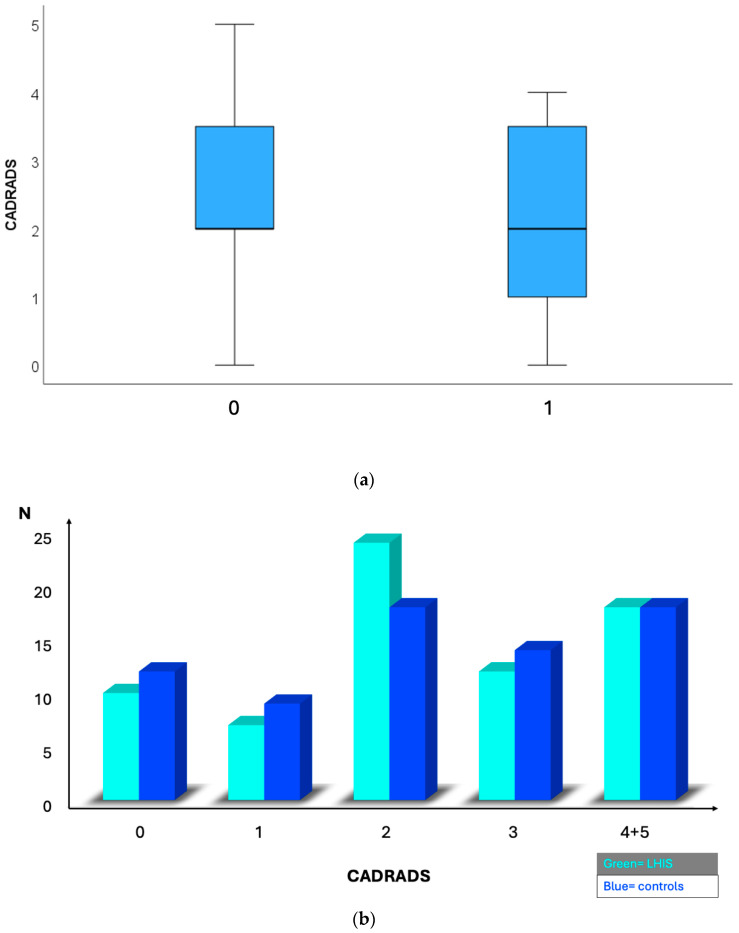
(**a**) Comparison of atherosclerosis features by CTA between LHIS and controls: There was no difference in coronary stenosis severity (CADRADS) between group 1 = LHIS vs. 0 = controls. (**b**) Coronary stenosis severity (CADRADS) in patients with LHIS (green) compared to controls (blue). N = counts. The non-obstructive disease (stenosis 25–49%, CADRADS 2) rate was not different in patients with LHIS vs. CR (33.8% vs. 25.4%, *p* = 0.358). There was no difference in coronary stenosis severit (CADRADS) and the obstructive disease rate (CADRADS 3+4+5) (stenosis > 50%), and no difference in plaque burden stratified according to CADRADS P0-4 classification (median 1.0 (IQR 1) and median 1.0 (IQR 1.0); *p* = 0.446).

Coronary artery disease (CAD) by coronary computed tomography (CTA): HRP (Figure 2) prevalence was significantly lower in the LHIS cohort than in the controls (21.1% vs. 40.8%; *p* < 0.011), while CAC (*p* = 0.827) and stenosis severity, according to CADRADS (*p* = 0.329), were not different. (Figure 3 and Figure 4a). The non-obstructive CAD (stenosis 25–49%, CADRADS 2) rate was slightly higher in patients with LHIS (24 (33.8%) vs. 18 (25.4%)); however, this was not significant (*p* = 0.358). There was no difference in the coronary stenosis severity scores (CADRADS) and obstructive disease rate (CADRADS 3+4+5) (stenosis > 50%) (Figure 4b)**.** SCORE2 was not different between LHIS and controls (mean: 36.65% +/− 1.30 SD for controls and 36.38% +/− 1.00 SD for LHIS). There was one CADRADS 2.0 exemption (E), a single coronary artery anomaly (LCA and RCA arising from right sinus valsalva). LHIS length and width by CTA: Mean width was 7.08 mm ± 1.6 (range: 3.8–12.5 mm), length was 32.3 ± 6.6 mm (range: 15–53 mm), and mean LHIS volume was mean 7.32 mL (range: 0.98–17.2 mL). The interobserver variability for HRP detection was high, with a weighted kappa of 0.91 (95% CI +/− 0.62, 95% CI 0.788–1.032, *p* > 0.001), and the intraobserver variability was similar (kappa 0.905 +/− 0.42; 95% CI 0.823–0.986, *p* > 0.001). In the LHIS group, 8 of 15 (53.3%) patients with HRP had two HRP features, 6 (40%) patients had three, and 1 (6.7%) of 15 patients had all four HRP criteria. In the control group, 13 of 29 (44.8%) patients with HRP had two HRP criteria, 10 (34.4%) patients had features, and 6 patients (20.7%) had four HRP features. The sum of HRP criteria was numerically higher in the control group vs. LHIS (80 vs. 40); however, ranked as score 2,3,4, it was not statistically different (median 3 (IQR 1) in CR vs. median 2 (IQR 1) in LHIS; Mann–Whitney U, *p* = 0.259).

Clinical Outcomes (Table 2): There was no difference in cardiovascular outcomes, with a low event rate for both death and ACS (*n* = 1 for each within each group). The rate of obstructive disease (>50% stenosis) by ICA and the revascularization rates (either CABG or PCI) between patients with LHIS and controls were not different. The total number of ICAs performed in each group was higher for controls (33.8% vs. 53.5%, *p* = 0.027). The mean follow up period was 3.74 years +/− 2.64 SD (range: 28 days up to 4524 days).

## 4. Discussion

Our study investigates the association between lipomatous hypertrophy of the interatrial septum (LHIS) and cardiovascular (CV) risk by evaluating the prevalence of high-risk plaque (HRP), the severity of coronary artery stenosis, and coronary artery calcification (CAC) using computed tomography angiography (CTA). Our findings reveal a significantly lower prevalence of HRP in the LHIS group compared to a matched control group. However, other less sensitive coronary artery disease (CAD) imaging parameters—including CAC score, coronary stenosis severity (CADRADS), and the prevalence of obstructive CAD—did not show significant differences between the two groups.

The key finding of our study is the significantly lower prevalence of HRP in patients with LHIS. HRP is a known predictor of future cardiovascular events [8] due to its association with plaque instability and an increased risk of acute coronary syndromes (ACS), such as myocardial infarction. The SCOT-Heart trial demonstrated a 4-fold increased risk of ACS in patients with HRP [8].

The lower HRP incidence in the LHIS group raises the hypothesis that LHIS may represent a valuable imaging biomarker for reduced cardiovascular (CV) risk. This finding could help identify a subset of patients who, despite exhibiting a considerable cardiovascular risk profile, as indicated by the SCORE2 in our cohort, present with less vulnerable coronary plaque. One possible explanation is the presence of brown adipose tissue (BAT) within LHIS, which may exert a protective effect. Unlike white adipose tissue, BAT is metabolically active and has been associated with improved lipid metabolism, enhanced metabolic health, and lower cardiovascular risk [20,21,22]. Nevertheless, our findings should be considered hypothesis-generating. Further studies in larger cohorts are required to determine whether a mechanistic link exists between brown fat within LHIS, the lower prevalence of HRP, and a potentially reduced overall CV risk.

The strength of our study encompasses a stringent propensity score matchmaking for all major cardiovascular risk factors including age, gender, BMI, smoking, positive family history, dyslipidemia, and diabetes. Furthermore, the baseline cardiovascular risk, as measured by SCORE2, was comparable between the groups.

Also, lipid panels cholesterol, c-LDL, HDL, and triglycerides were not different between both groups. Elevated c-LDL is a predisposing factor for lipid-rich non-calcified low attenuation plaque (LAP) [23], while a higher HDL acts protective, with lower HDL being associated with higher coronary plaque burden [24]. In the Miami heart trial [25], a large cohort study enrolling 1808 statin-naïve asymptomatic patients, a positive trend between c-LDL and any plaque (17.9–45.2%) or non-calcified plaque (12.8–23.8%) was observed in the low-risk group.

Further, there was no difference in statin intake between both groups in our cohort. Statins increase plaque density and reduce the lipid rich plaque component, by triggering the calcification pathway, reduce plaque progression and high-risk plaque features [26,27,28]. Coronary plaque with a higher CT density up to 1000 Hounsfield units (“1k” plaque) is associated with a lower likelihood of acute coronary syndromes [29], and a recent metanalysis confirmed the decreased MACE rate in patients with higher calcium density [30].

Despite the lower HRP prevalence, we found no differences in the total coronary calcium burden expressed by the CAC score, total visual plaque burden by the CADRADS P0-4 classification and coronary stenosis severity (CADRADS) grading, and rates of obstructive disease. While CAC is an accurate tool for quantifying the calcium burden and total plaque burden, it does not take into account the non-calcifying plaque (NCP) component, consisting of lipid-rich and fibrous plaques. Novel data show that these features of atherosclerosis (including NCP and total plaque burden and stenosis severity), in combination with other CT parameters, via artificial intelligence (AI)-quantitative CT (AI-QCT) [7,31,32], provide more accurate and nuanced prognostic parameters than conventional methods [31], while visual scoring of stenosis has a moderate interobserver agreement [33] due to high inter-reader variability [34].

This is consistent with the observation that cardiovascular outcomes, including death, rates of ICA stenosis > 50%, revascularization, and ACS, were comparable between the LHIS and control groups. However, the sample size of our study cohort was very small, and event rates were too low for definite conclusions on CV outcomes.

In summary, our study results suggest that LHIS may indicate the qualitative nature of atherosclerosis (i.e., less vulnerable “high-risk” plaque), without an impact of less sensitive markers such as stenosis severity and the CAC.

The lack of significant differences in CAC scores between groups may reflect the limitations of CAC as a marker of coronary risk. While CAC is a well-established measure of atherosclerotic burden, it predominantly reflects calcified plaque and does not capture the total plaque burden [7] and other plaque phenotypes such as non-calcified (NCP), lipid-rich “high-risk” HRP [8], or % percentage atheroma volume fibrous, which allow for a more nuanced CV risk stratification [32]. HRP represents a more specific marker for vulnerable plaque [8], which may explain our study findings.

Study Limitations: First, as we investigated a novel imaging biomarker that has not been previously studied, our sample size was relatively small. In particular, the number of major adverse cardiovascular events (MACE)—especially death and myocardial infarction—was low, and too low to allow for a statistically powered outcome analysis. Second, the retrospective design of the study introduces inherent biases that must be acknowledged.

Third, there were few (*n* = 3) patients after PCI included. A further limitation is the absence of PET/CT imaging to confirm the metabolic activity or the presence of brown adipose tissue in LHIS. Although prior studies have suggested BAT-like characteristics in LHIS with PET-tracer uptake [35], this remains speculative in our cohort and cannot be conclusively proven without metabolic imaging. Finally, the majority of patients was male (94%); hence, the dataset is not representative for women.

## 5. Conclusions

In conclusion, this study highlights the potential of LHIS as a novel imaging biomarker for reversing cardiovascular risk due to its association with a lower prevalence of high-risk plaque.

### Clinical Translation Statement

LHIS may act as a reverse cardiovascular prognosticator, guiding a more personalized risk stratification for patients with suspected coronary artery disease beyond EAT, CAC, and other established CT imaging biomarkers such as plaque phenotypes [8,31,32] and conventional tools.

## Figures and Tables

**Table 1:** Study cohort: demographics, cardiovascular risk factors (CVRF), CTA results, and medication.

	LHIS N = 71	Controls N = 71	
Age (yrs)	61.1 ± 9.9	60.6 ± 11.4	0.754
women	5 (7%)	4 (5.6%)	0.999
BMI kg/m^2^	28.9 ± 4.4	27.6 ± 4.8	0.096
AFIB	7 (9.8%)	12 (16.9%)	0.324
COPD	5 (7%)	4 (5.6%)	0.999 *
**CVRF**			
Smoking	34 (47.9%)	35 (49.3%)	0.999
Arterial hypertension	40 (56.3%)	33 (46.5%)	0.316
Positive Family History	26 (36.6%)	29 (40.8%)	0.730
Dyslipidemia	41 (57.7%)	38 (53.5%)	0.612
Diabetes	19 (26.7%)	16 (22.5%)	0.559
**CTA results**			
HRP	15 (21.1%)	29 (40.8%)	0.011
CAC (AU)	Mean 291.86 ± 565 Median 32.8 (IQR 152.5)	Mean 186.1 ± 408 Median 44.9 (IQR 316.3)	0.827
CADRADS	Mean 2.23 ± 0.17	Mean 2.30 ± 0.16	0.329
	Median 2 (IQR 3)	Median 2 (IQR 2)	
0	10 (14.1%)	12 (16.9%)	
1	7 (9.9%)	9 (12.7%)	
2	24 (33.8%)	18 (25.4%)	
3	12 (16.9%)	14 (19.7%)	
4	17 (23.9%)	18 (25.45)	0.358
5	1 (1.4%)	0 (0%)	
Obstructive disease rate (>50% stenosis)	30 (42.3%)	32 (45.1%)	0.866
**Medication**			
**Statins**	35 (49.3%)	26 (36.6%)	0.136
**Lipid panels**			
Cholesterol (mg/dL)	190.15 ± 43.4	191.7 ± 49.02	0.868
c-LDL (mg/dL)	123.8 ± 41.6	117.2 ± 44.5	0.479
HDL (mg/dL)	52.1 ± 14.9	53.8 ± 17.9	0.611
TG (mg/dL)	130.7 ± 10.9	158.6 ± 18.6	0.309

Abbreviations: LHIS = lipomatous hypertrophy of the interatrial septum; BMI = body mass index; CAC = coronary artery calcium score; CTA = computed tomography angiography; AFIB = atrial fibrillation; COPD = chronic obstructive pulmonary disease; HRP = high-risk plaque; IQR = interquartile range; AU = Agatston units; CVRF = cardiovascular risk factors. * Fisher’s exact.

**Table 2:** Clinical outcomes (MACE—death and myocardial infarcts (ACS), coronary revascularization rate).

	LHIS N = 71	Controls N = 71	
ICA N (%)	24 (33.8%)	38 (53.5%)	0.027
ICA > 50%	19 (26.7%)	17 (23.9%)	0.847
Revascularization rate	17 (23.9%)	11 (15.5%)	0.293
	N = 13 PCI	N = 11 PCI	
	N = 4 CABG	N = 0 CABG	
Death	1 MCI (CV death)	1 (* non-cardiac)	0.999
ACS (STEMI or NSTEMI)	1 (NSTEMI)	1 (STEMI)	0.999

Abbreviations: ICA = invasive coronary angiography; c-LDL = low-density lipoprotein; HDL = high-density lipoprotein; TG = triglycerids; ACS = acute coronary syndromes; MCI = myocardial infarct; N = counts; PCI = percutaneous coronary intervention; CABG = coronary artery bypass grafting; MACE = major adverse cardiovascular events. * non-cardiac death (organ failure due to autoimmune disease—Sjögren Syndrome).

## Data Availability

The original contributions presented in this study are included in the article. Further inquiries can be directed to the corresponding author.

## Abbreviations

The following abbreviations are used in this manuscript:

LHIS	lipomatous hypertrophy of the interatrial septum
CTA	computed tomography angiography
CAD	coronary artery disease
HRP	high-risk plaque
CAC	coronary artery calcium score
HU	Hounsfield units
CR	controls
CV	cardiovascular

## References

[1] Martin S.S., Aday A.W., Allen N.B., Almarzooq Z.I., Anderson C.A.M., Arora P., Avery C.L., Baker-Smith C.M., Bansal N., Beaton A.Z. (2025). 2025 Heart Disease and Stroke Statistics: A Report of US and Global Data From the American Heart Association. Circulation.

[2] Rana J.S., Tabada G.H., Solomon M.D., Lo J.C., Jaffe M.G., Sung S.H., Ballantyne C.M., Go A.S. (2016). Accuracy of the Atherosclerotic Cardiovascular Risk Equation in a Large Contemporary, Multiethnic Population. J. Am. Coll. Cardiol..

[3] SCORE2 Working Group, ESC Cardiovascular Risk Collaboration (2021). SCORE2 risk prediction algorithms: New models to estimate 10-year risk of cardiovascular disease in Europe. Eur. Heart J..

[4] Blaha M.J., Cainzos-Achirica M., Dardari Z., Blankstein R., Shaw L.J., Rozanski A., Rumberger J.A., Dzaye O., Michos E.D., Berman D.S. (2020). All-cause and cause-specific mortality in individuals with zero and minimal coronary artery calcium: A long-term, competing risk analysis in the Coronary Artery Calcium Consortium. Atherosclerosis.

[5] van Rosendael A.R., Shaw L.J., Xie J.X., Dimitriu-Leen A.C., Smit J.M., Scholte A.J., van Werkhoven J.M., Callister T.Q., DeLago A., Berman D.S. (2019). Superior Risk Stratification With Coronary Computed Tomography Angiography Using a Comprehensive Atherosclerotic Risk Score. JACC Cardiovasc. Imaging.

[6] Xie J.X., Cury R.C., Leipsic J., Crim M.T., Berman D.S., Gransar H., Budoff M.J., Achenbach S., Ó Hartaigh B., Callister T.Q. (2018). The Coronary Artery Disease-Reporting and Data System (CAD-RADS): Prognostic and Clinical Implications Associated With Standardized Coronary Computed Tomography Angiography Reporting. JACC Cardiovasc. Imaging.

[7] Nurmohamed T.P.V., Nurmohamed N.S., Min J.K., Anthopolos R., Reynolds H.R., Earls J.P., Crabtree T., Mancini G.B.J., Leipsic J., Budoff M.J. (2024). Atherosclerosis quantification and cardiovascular risk: The ISCHEMIA trial. Eur. Heart J..

[8] Williams M.C., Kwiecinski J., Doris M., McElhinney P., D’Souza M.S., Cadet S., Adamson P.D., Moss A.J., Alam S., Hunter A. (2020). Low-Attenuation Noncalcified Plaque on Coronary Computed Tomography Angiography Predicts Myocardial Infarction: Results From the Multicenter SCOT-HEART Trial (Scottish Computed Tomography of the HEART). Circulation.

[9] Goeller M., Achenbach S., Marwan M., Doris M.K., Cadet S., Commandeur F., Chen X., Slomka P.J., Gransar H., Cao J.J. (2018). Epicardial adipose tissue density and volume are related to subclinical atherosclerosis, inflammation and major adverse cardiac events in asymptomatic subjects. J. Cardiovasc. Comput. Tomogr..

[10] Oikonomou E.K., Antonopoulos A.S., Schottlander D., Marwan M., Mathers C., Tomlins P., Siddique M., Klüner L.V., Shirodaria C., Mavrogiannis M.C. (2021). Standardized measurement of coronary inflammation using cardiovascular computed tomography: Integration in clinical care as a prognostic medical device. Cardiovasc. Res..

[11] Lacaita P.G., Senoner T., Bilgeri V., Rauch S., Barbieri F., Kindl B., Plank F., Dichtl W., Deeg J., Widmann G. (2024). The interaction of lipomatous hypertrophy of the interatrial septum with pericardial adipose tissue biomarkers by computed tomography. Eur. Radiol..

[12] Fan C.M., Fischman A.J., Kwek B.H., Abbara S., Aquino S.L. (2005). Lipomatous hypertrophy of the interatrial septum: Increased uptake on FDG PET. Am. J. Roentgenol..

[13] Becher T., Palanisamy S., Kramer D.J., Eljalby M., Marx S.J., Wibmer A.G., Butler S.D., Jiang C.S., Vaughan R., Schöder H. (2021). Brown adipose tissue is associated with cardiometabolic health. Nat. Med..

[14] Aldiss P., Davies G., Woods R., Budge H., Sacks H.S., Symonds M.E. (2017). ‘Browning’ the cardiac and peri-vascular adipose tissues to modulate cardiovascular risk. Int. J. Cardiol..

[15] Knuuti J., Wijns W., Saraste A., Capodanno D., Barbato E., Funck-Brentano C., Prescott E., Storey R.F., Deaton C., Cuisset T. (2020). ESC Scientific Document Group. 2019 ESC Guidelines for the diagnosis and management of chronic coronary syndromes. Eur. Heart J..

[16] Agatston A.S., Janowitz W.R., Hildner F.J., Zusmer N.R., Viamonte M., Detrano R. (1990). Quantification of coronary artery calcium using ultrafast computed tomography. J. Am. Coll. Cardiol..

[17] Cury R.C., Abbara S., Achenbach S., Berman D., Bittencourt M., Budoff M., Chinnaiyan K., Choi A.D., Ghoshhajra B., Jacobs J. (2016). CAD-RADS^TM^: Coronary Artery Disease—Reporting and Data System: An Expert Consensus Document of the Society of Cardiovascular Computed Tomography (SCCT), the American College of Radiology (ACR) and the North American Society for Cardiovascular Imaging (NASCI). Endorsed by the American College of Cardiology. J. Am. Coll. Radiol..

[18] Maurovich-Horvat P., Schlett C.L., Alkadhi H., Nakano M., Otsuka F., Stolzmann P., Scheffel H., Ferencik M., Kriegel M.F., Seifarth H. (2012). The napkin-ring sign indicates advanced atherosclerotic lesions in coronary CT angiography. JACC Cardiovasc. Imaging.

[19] Williams B., Mancia G., Spiering W., Agabiti Rosei E., Azizi M., Burnier M., Clement D.L., Coca A., De Simone G., Dominiczak A. (2018). 2018 ESC/ESH Guidelines for the management of arterial hypertension. Eur. Heart J..

[20] Societies E.N.C., Mach F., Baigent C., Taskinen M.-R. (2019). 2019 ESC/EAS guidelines for the management of dyslipidaemias: Lipid modification to reduce cardiovascular risk. Atherosclerosis.

[21] Cosentino F., Grant P.J., Aboyans V., Bailey C.J., Ceriello A., Delgado V., Federici M., Filippatos G., Grobbee D.E., Hansen T.B. (2020). 2019 ESC Guidelines on diabetes, pre-diabetes, and cardiovascular diseases developed in collaboration with the EASD. Eur. Heart J..

[22] Page D.L. (1970). Lipomatous hypertrophy of the cardiac interatrial septum. Hum. Pathol..

[23] Hagan K., Mszar R., Cainzos-Achirica M., Blaha M.J., Shapiro M.D., Arias L., Saxena A., Cury R., Budoff M.J., Feldman T. (2024). Low-density lipoprotein-cholesterol and subclinical coronary atherosclerosis in a middle-aged asymptomatic U.S. population: The Miami Heart Study at Baptist Health South Florida. Atherosclerosis.

[24] Manubolu V.S., Verghese D., Lakshmanan S., Alalawi L., Kinninger A., Bitar J.A., Calicchio F., Ahmad K., Ghanem A., Javier D.A. (2022). Coronary computed tomography angiography evaluation of plaque morphology and its relationship to HDL and total cholesterol to HDL ratio. J. Clin. Lipidol..

[25] Banach M., Serban C., Sahebkar A., Mikhailidis D.P., Ursoniu S., Ray K.K., Rysz J., Toth P.P., Muntner P., Mosteoru S. (2015). Lipid and Blood Pressure Meta-analysis Collaboration (LBPMC) Group. Impact of statin therapy on coronary plaque composition: A systematic review and meta-analysis of virtual histology intravascular ultrasound studies. BMC Med..

[26] Lee S.E., Chang H.J., Sung J.M., Park H.B., Heo R., Rizvi A., Lin F.Y., Kumar A., Hadamitzky M., Kim Y.J. (2018). Effects of Statins on Coronary Atherosclerotic Plaques: The PARADIGM Study. JACC Cardiovasc. Imaging.

[27] Park H.B., Arsanjani R., Sung J.M., Heo R., Lee B.K., Lin F.Y., Hadamitzky M., Kim Y.J., Conte E., Andreini D. (2023). Impact of statins based on high-risk plaque features on coronary plaque progression in mild stenosis lesions: Results from the PARADIGM study. Eur. Heart J. Cardiovasc. Imaging.

[28] Puri R., Nicholls S.J., Shao M., Kataoka Y., Uno K., Kapadia S.R., Tuzcu E.M., Nissen S.E. (2015). Impact of statins on serial coronary calcification during atheroma progression and regression. J. Am. Coll. Cardiol..

[29] van Rosendael A.R., Narula J., Lin F.Y., van den Hoogen I.J., Gianni U., Al Hussein Alawamlh O., Dunham P.C., Peña J.M., Lee S.E., Andreini D. (2020). Association of High-Density Calcified 1K Plaque With Risk of Acute Coronary Syndrome. JAMA Cardiol..

[30] Yong Y., Giovannucci J., Pang S.N., Hong W., Han D., Berman D.S., Dey D., Nicholls S.J., Nerlekar N., Lin A. (2024). Coronary Artery Calcium Density and Risk of Cardiovascular Events: A Systematic Review and Meta-Analysis. JACC Cardiovasc. Imaging.

[31] Stuijfzand W.J., van Rosendael A.R., Lin F.Y., Chang H.J., van den Hoogen I.J., Gianni U., Choi J.H., Doh J.H., Her A.Y., Koo B.K. (2020). CREDENCE Investigators. Stress Myocardial Perfusion Imaging vs Coronary Computed Tomographic Angiography for Diagnosis of Invasive Vessel-Specific Coronary Physiology: Predictive Modeling Results From the Computed Tomographic Evaluation of Atherosclerotic Determinants of Myocardial Ischemia (CREDENCE) Trial. JAMA Cardiol..

[32] Feuchtner G.M., Lacaita P.G., Bax J.J., Rodriguez F., Nakanishi R., Pontone G., Mushtaq S., Buechel R.R., Gräni C., Patel A.R. (2025). AI-Quantitative CT Coronary Plaque Features Associate with a Higher Relative Risk in Women: CONFIRM2-Registry. Circ. Cardiovasc. Imaging.

[33] Rovetto C., Niemann T., Almeida G., Aghapour Zangeneh F., Chikh Bakri I., Sartoretti T., Halfmann M.C., Emrich T., Euler A. (2025). Assessment of inter- and intrareader agreement among different stenosis quantification methods in coronary CT angiography. Eur. J. Radiol..

[34] Bernardo R., Nurmohamed N.S., Bom M.J., Jukema R., de Winter R.W., Sprengers R., Stroes E.S.G., Min J.K., Earls J., Danad I. (2025). Diagnostic accuracy in coronary CT angiography analysis: Artificial intelligence versus human assessment. Open Heart.

[35] Kuester L.B., Fischman A.J., Fan C.M., Halpern E.F., Aquino S.L. (2005). Lipomatous hypertrophy of the interatrial septum: Prevalence and features on fusion 18F fluorodeoxyglucose positron emission tomography/CT. Chest.

